# Multiple machine learning-based integrations of multi-omics data to identify molecular subtypes and construct a prognostic model for HNSCC

**DOI:** 10.1186/s41065-025-00380-0

**Published:** 2025-02-06

**Authors:** Xiaoqin Luo, Chao Li, Gang Qin

**Affiliations:** 1 Department of Otolaryngology, University of Electronic Science and Technology of China, Chengdu, 611731 China; 2 Department of Otolaryngology, The Affiliated Hospital, Southwest Medical University, Luzhou, 646000 China; 3 Department of Otolaryngology, The Affiliated Traditional Chinese Medicine Hospital, Southwest Medical University, Luzhou, 646000 China; 4 Head and Neck Surgery Department, Sichuan Clinical Research Center for Cancer, Sichuan Cancer Hospital & Institute, Sichuan Cancer Center, Affiliated Cancer Hospital of University of Electronic Science and Technology of China, Chengdu, China

**Keywords:** Head and neck squamous cell carcinoma, Multi-omics analyses, Machine learning, Prognostic model, Immunotherapy

## Abstract

**Background:**

Immunotherapy has introduced new breakthroughs in improving the survival of head and neck squamous cell carcinoma (HNSCC) patients, yet drug resistance remains a critical challenge. Developing personalized treatment strategies based on the molecular heterogeneity of HNSCC is essential to enhance therapeutic efficacy and prognosis.

**Methods:**

We integrated four HNSCC datasets (TCGA-HNSCC, GSE27020, GSE41613, and GSE65858) from TCGA and GEO databases. Using 10 multi-omics consensus clustering algorithms via the MOVICS package, we identified two molecular subtypes (CS1 and CS2) and validated their stability. A machine learning-driven prognostic signature was constructed by combining 101 algorithms, ultimately selecting 30 prognosis-related genes (PRGs) with the Elastic Net model. This signature was further linked to immune infiltration, functional pathways, and therapeutic sensitivity.

**Results:**

CS1 exhibited superior survival outcomes in both TCGA and META-HNSCC cohorts. The PRG-based signature stratified patients into low- and high-risk groups, with the low-risk group showing prolonged survival, enhanced immune cell infiltration (B cells, T cells, monocytes), and activated immune functions (cytolytic activity, T cell co-stimulation). High-risk patients were more sensitive to radiotherapy and chemotherapy (e.g., Cisplatin, 5-Fluorouracil), while low-risk patients responded better to immunotherapy and targeted therapies.

**Conclusion:**

Our study delineates two molecular subtypes of HNSCC and establishes a robust prognostic model using multi-omics data and machine learning. These findings provide a framework for personalized treatment selection, offering clinical insights to optimize therapeutic strategies for HNSCC patients.

**Supplementary Information:**

The online version contains supplementary material available at 10.1186/s41065-025-00380-0.

## Introduction

Head and neck squamous cell carcinoma (HNSCC), a heterogeneous malignancy arising from mucosal epithelia in the oral cavity, pharynx, and larynx, remains a significant global health challenge due to its aggressive behavior and poor prognosis. According to GLOBOCAN 2020, HNSCC accounts for over 830,000 new cases and 430,000 deaths annually worldwide, ranking seventh in cancer incidence and mortality [1, 2]. In the United States alone, an estimated 54,540 new cases and 11,580 deaths were projected for 2023 [3]. The disease exhibits a striking gender disparity, with males facing a 2.5-fold higher incidence than females, driven by established risk factors such as tobacco use, alcohol consumption, and high-risk human papillomavirus (HPV) infection [4, 5]. While HPV-positive oropharyngeal cancers often respond better to therapy, the majority of HNSCC patients present with advanced-stage disease characterized by high rates of locoregional recurrence, distant metastasis, and 5-year survival rates below 50% despite multimodal therapies [6–8].

Current standard treatments—surgery, radiotherapy, and platinum-based chemotherapy—are associated with severe toxicities, including dysphagia, mucositis, and irreversible organ dysfunction, which profoundly impair patients’ quality of life [9, 10]. The advent of immune checkpoint inhibitors (ICIs) targeting PD-1/PD-L1 (e.g., pembrolizumab, nivolumab) and CTLA-4 (e.g., tremelimumab) has revolutionized HNSCC management, offering durable responses in a subset of patients by reactivating antitumor immunity [11–13]. However, clinical benefits remain limited, with objective response rates below 20% in recurrent/metastatic settings, and intrinsic or acquired resistance mechanisms—such as T cell exhaustion, immunosuppressive myeloid cell infiltration, and defects in antigen presentation—continue to hinder therapeutic efficacy [14–16]. These challenges underscore the critical need for strategies to predict treatment responsiveness and tailor therapies based on molecular profiles.

Advances in multi-omics technologies (genomics, transcriptomics, epigenomics) have unraveled the profound molecular heterogeneity of HNSCC, revealing distinct subtypes defined by HPV status, somatic mutations (e.g., TP53, CDKN2A, PIK3CA), and immune microenvironment composition [17–19]. Similarly, transcriptomic analyses have identified immune “hot” and “cold” tumors, with the former showing enhanced cytotoxic lymphocyte infiltration and superior responses to ICIs [21]. Despite these insights, translating molecular subtyping into clinically actionable frameworks remains elusive, partly due to the lack of robust prognostic models integrating multi-omics data and real-world treatment outcomes.

Machine learning (ML) has emerged as a powerful tool to address this gap, enabling the integration of high-dimensional omics data for predictive modeling. Recent studies have leveraged ML algorithms to identify prognostic gene signatures, predict drug sensitivity, and stratify patients for immunotherapy [22–24]. However, many models suffer from overfitting, limited generalizability, or a failure to account for the dynamic interplay between tumor cells and the immune microenvironment. Furthermore, most existing signatures rely on single-omics approaches, neglecting the complementary insights offered by multi-omics integration [25].

In this study, we integrated transcriptomic, genomic, and clinical data from four independent HNSCC cohorts to define molecular subtypes and construct a machine learning-driven prognostic signature. By employing consensus clustering algorithms and ML models, we identified two robust subtypes (CS1 and CS2) and a 30-gene prognostic signature linked to immune infiltration and therapeutic sensitivity. These findings advance the paradigm of precision oncology in HNSCC, offering actionable insights to optimize therapeutic strategies and improve patient outcomes.

## Materials and methods

### Downloading and processing of raw data

We collected HNSCC datasets from the TCGA database, GEO database, and previous literature based on the following criteria: HNSCC (including oral cavity, pharynx, and larynx, etc.) as the research direction, the inclusion of prognostic information, a minimum dataset size of over 80 cases, and maximal gene similarity between datasets. As a result, we collected four datasets: TCGA-HNSCC, GSE27020, GSE41613, and GSE65858 (Table 1). We acquired omics data on HNSCC from the TCGA-HNSCC cohort, including mRNA, lncRNA, microRNA, DNA methylation, somatic mutations, and clinical data. We only retained 489 samples that contained both the above histologic data and clinical data. For mRNA and lncRNA data, we used log2 (TPM + 1) to make the data more normally distributed. For microRNA data, we used log2 (RPM + 1) to make the data more normally distributed. In addition, we acquired microarray and clinical data for three HNSCC cohorts from GEO (GSE27020, GSE41613, and GSE65858) [18–21]. For the microarray data, we use log2 ((RMA or RSN) + 1) to make the data more normally distributed. Finally, we used the “ComBat” function from the “sva” package, which uses a parametric empirical Bayesian framework to adjust the batch effect data, merging the different queues to eliminate the batch effect [22].

### Multiomics consensus clustering analysis

The first step was to identify elite genes via the “getElites” function from the “MOVICS” package, which aims to reduce data dimension for multi-omics integrative clustering analysis [23]. For continuous variables (mRNA, lncRNA, microRNA, and DNA methylation data), we selected the top 1500 genes with the highest degree of variation by setting the “mad” parameter and used univariate Cox analysis to identify prognostic-related genes (PRGs) in each cohort (*p* < 0.05). For discrete variables (somatic mutation data), we selected the top 5% of genes with the highest mutation frequency by setting the “freq” parameter [24]. The optimal number of clusters was determined by evaluating the sum of the Clustering Prediction Index (CPI) and gap statistics [25]. Next, we obtained the optimal number of clusters with the “getClustNum” function from the “MOVICS” package and performed cluster analysis with the “getMOIC” function from the “MOVICS” package, which contains 10 clustering algorithms (SNF, CIMLR, PINSPlus, NEMO, COCA, MoCluster, LRAcluster, ConsensusClustering, IntNMF, and iClusterBayes) [25–28]. Lastly, we borrowed the idea of consensus clustering to integrate the clustering results of multiple algorithms to improve the reliability and stability of the clustering.

### Validation of molecular subtypes

We utilized the “runMarker” function to identify marker genes for each subtype, specifying a threshold of n.marker = 1000 and adjusting the p-value cutoff to p.adj.cutoff = 0.05. To ascertain the robustness of the identified subtypes, we validated the clustering results by leveraging the marker genes specific to each subtype within the verification set. Additionally, we compared these results with the consistency achieved by the Nearest Template Prediction (NTP) and Partition Around Medoids (PAM) classifiers, employing them for consensus clustering. This comprehensive approach ensured that the subtypes were not only well-defined but also consistent across different validation methods.

### Gene expression profile, enrichment analysis, and immune cell infiltration

Firstly, we utilized Principal Component Analysis (PCA), T-distributed Stochastic Neighbor Embedding (TSNE), and Uniform Manifold Approximation and Projection (UMAP) analysis to find the gene distribution of cancer subtypes (CSs) [29]. We analyzed the gene expression patterns of genes in the follow-up model across different CSs, aiming to identify distinct regulatory patterns. Finally, we performed Gene Set Variation Analysis (GSVA) to evaluate the activation status of the Kyoto Encyclopedia of Genes and Genomes (KEGG) pathway in each CS and evaluated the level of immune cell infiltration in different CSs using the single-sample Gene Set Enrichment Analysis (ssGSEA) method [30].

### Development of a prognostic signature

Given the limited sample size in some cohorts, we pooled the three cohorts together to form the META-HNSCC cohort. Aiming to develop a prognostic model with high accuracy and generalizability, we integrated 10 diverse machine learning methods, including CoxBoost, stepwise Cox, Lasso, Ridge, Enet, survival-SVMs, GBMs, SuperPC, plsRcox, and RSF [31–34]. The development pipeline of the machine learning-driven signature is as follows. First, we performed univariate Cox analysis within the TCGA-HNSCC and META-HNSCC datasets. Genes with *p* < 0.05 and the same hazard ratio (HR) orientation were considered PRGs. Then, 101 combinations of 10 machine learning algorithms were utilized to develop the most predictive signature with the best C-index performance. Finally, upon establishing the model on the training set, we proceeded to rigorously test it across all validation cohorts. For each model, we computed the average C-index, ultimately considering the model with the highest C-index value as the optimal one.

### Prognostic value and clinical application

We predicted the risk score using the linear predictor method based on the “predict” function in the “stats” package. We selected the best cutoff based on the “surv_cutpoint” function in the “survminer” package to classify into high- and low-risk groups. Survival analyses were conducted among these distinct groups to evaluate the prognostic significance of the signature. Additionally, we conducted a search for 19 prognostic signatures pertinent to HNSCC and computed scores for each sample [35–53]. The predictive capability of each signature within each cohort was assessed using the C-index. To compare the predictive power of the model with traditional clinical characteristics, the C-index was employed. Finally, we constructed a nomogram containing traditional clinical information and this model to predict the survival of HNSCC patients.

### Exploration of molecular functions, pathways, and gene mutations

We obtained differentially expressed genes (DEGs) in different risk groups by differential expression analysis (|log2FC > = 1| and FDR < 0.05) using the R package “limma”. Then, Gene Ontology (GO) and KEGG analyses were performed on the identified DEGs to clarify the biological functions and signaling pathways (*p* < 0.05) [34, 54, 55]. To delve deeper into the genomic intricacies of HNSCC and investigate its correlation with varying risk scores, the R package “Maftools” was employed [56].

### Exploration of the immune landscape

Various algorithms were utilized to deconvolve and quantify the immune cell composition within the tumor microenvironment (TME) [57–62]. To further delve into the immune function of the distinct groups, the ssGSEA approach was utilized. In addition, we analyzed the expression patterns of ICGs in distinct groups. Furthermore, the TIDE approach was used to anticipate the immunogenicity of HNSCC, thereby facilitating informed decisions regarding immunotherapy [63]. TIDE was a computational framework developed to evaluate the potential of tumor immune escape from the gene expression profiles of cancer samples.

### Prediction of treatment sensitivity

Apart from immunotherapy, chemotherapy and targeted therapies are conventional treatments for cancer. We used the ssGSEA algorithm to calculate two radiotherapy biomarkers to predict response to radiotherapy. Finally, the package “oncoPredict” was employed to forecast the sensitivity of patients in various groups to conventional chemotherapeutic drugs (5-Fluorouracil, Cisplatin, Docetaxel, Oxaliplatin, and Paclitaxel) and targeted therapy drugs [64]. The package “oncoPredict” allowed for building drug response models using screening data between bulk RNA-Seq and a drug response metric.

### Exploration of scRNA

We utilized the TISCH2 database to meticulously analyze the expression patterns of the signature genes in distinct cell populations isolated from HNSCC patients enrolled in the GSE103322 study [65]. This approach allowed us to delve into the TME of HNSCC at a single-cell resolution. The TISCH2 database serves as a valuable repository for the interpretation of scRNA-seq data, offering comprehensive gene expression profiles of diverse immune cells within the TME. We aimed to gain deeper insights into the potential involvement of distinct immune cell subsets in the pathogenesis and progression of HNSCC [66].

### Quantitative RT-PCR analysis

The np69 and FaDu cell line were obtained from Wuhan Pricella Biotechnology Co.,Ltd. The np69 and FaDu cell lines were cultured in DMEM. Total RNA was extracted from the cells using the TRIzol reagent (Invitrogen). A PrimeScript™ RT Reagent Kit (Takara, Japan) was used for reverse transcription, and PowerUp SYBR Green Master Mix (Thermo Fisher Scientifc) was used to perform qRT‒ PCR according to the manufacturer’sinstructions. The primer sequences are shown in Table S5.

## Results

### Recognition of multiple cancer subtypes

**Fig. 1:**
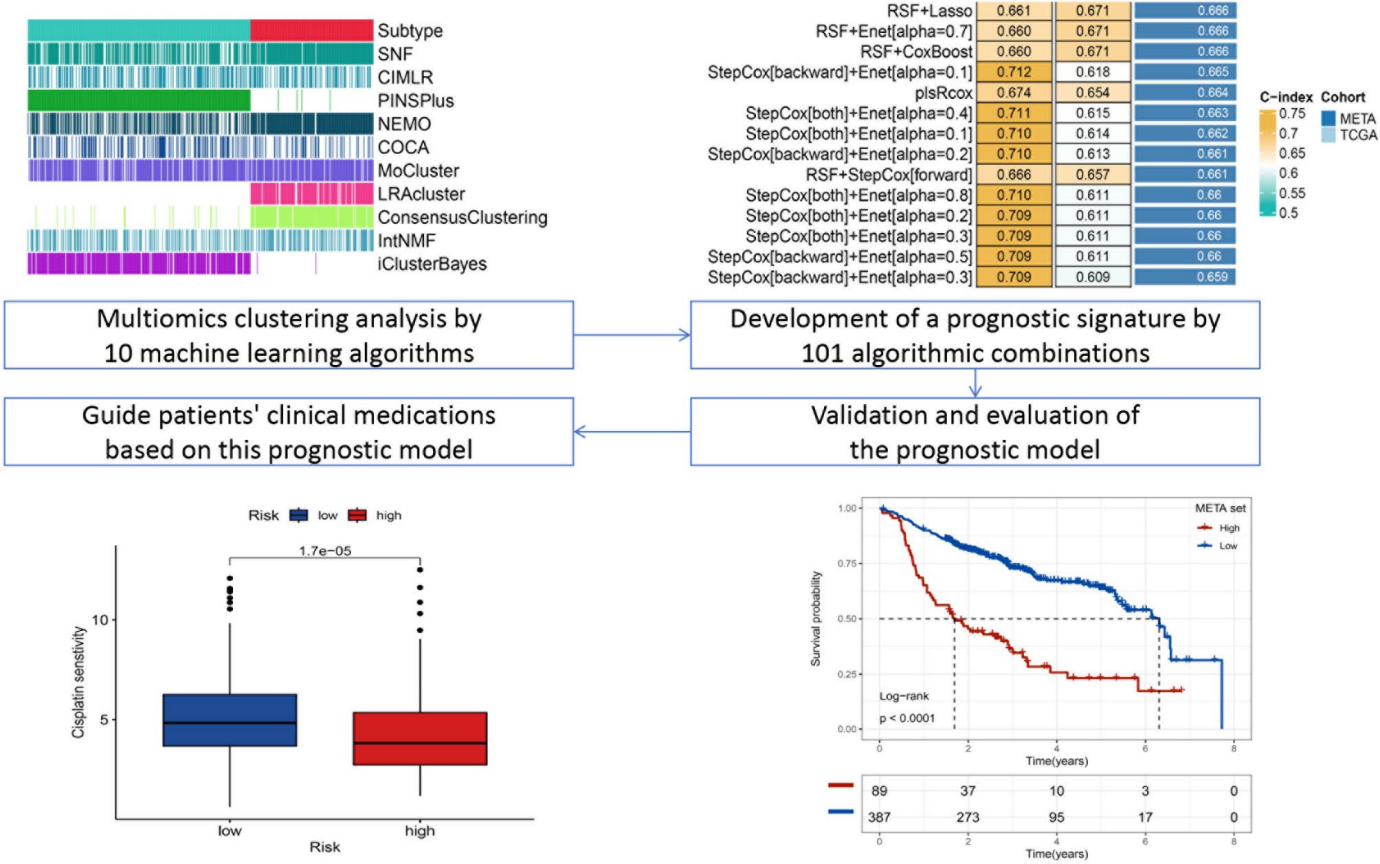
Flowchart of the article

**Fig. 2:**
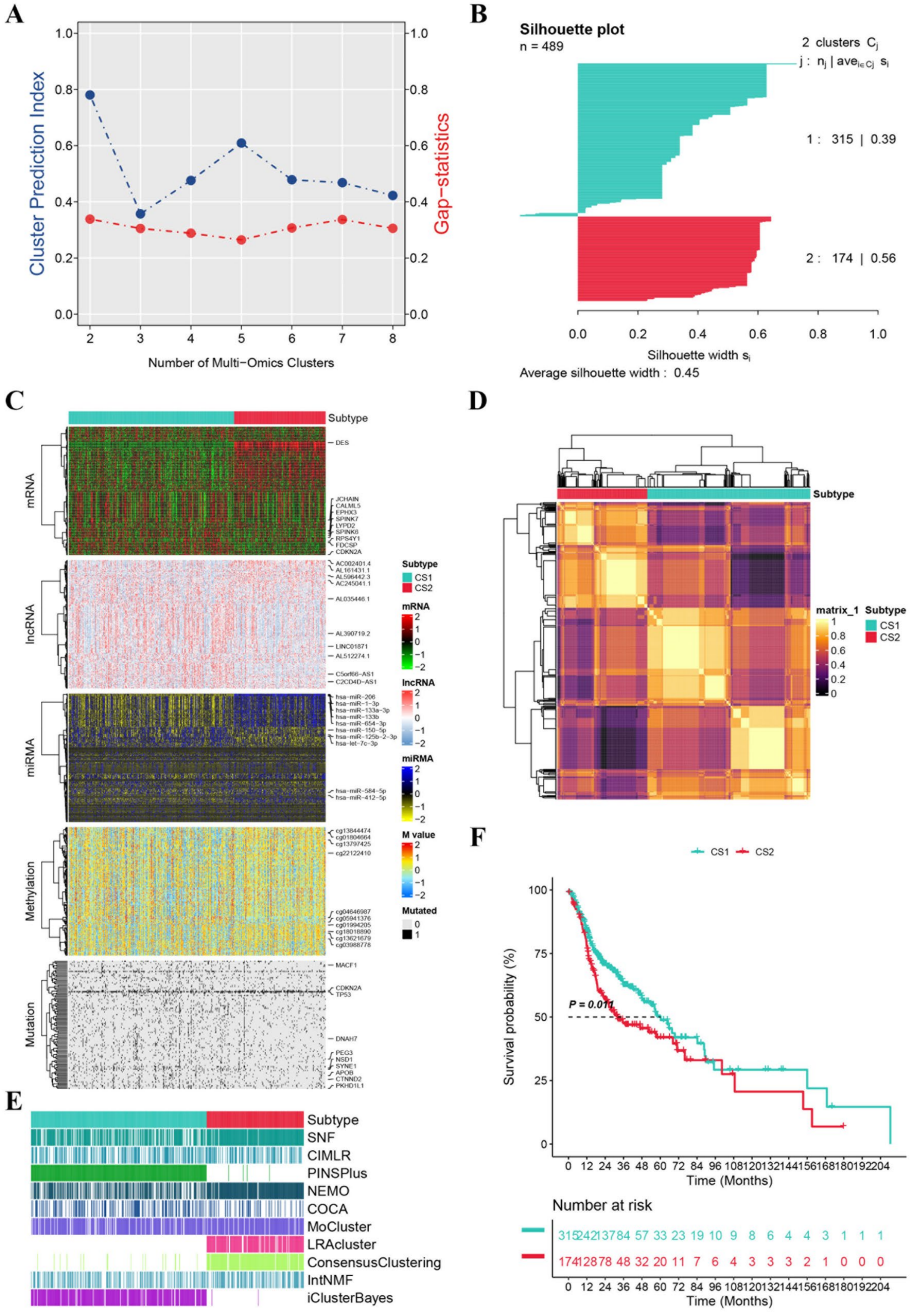
Subtypes of HNSCC based on multi-omics. (**A**) Calculation of the cluster prediction index and gap statistic to select the multi-omics clustering for HNSCC; (**B**) Calculating of the Silhoutte score to evaluate the sample similarity in each subgroup; (**C**) Visualization of multi-omics data; (**D**) Consensus heatmap for two clusters by the 10 multi-omics algorithms; (**F**) Kaplan-Meier analysis of OS in the two cluster subtypes

The workflow of our research is outlined in Fig. 1. We validated our findings by conducting a comparative analysis of the data both before and after addressing batch effects using PCA (Fig. S1A and B). Utilizing 10 multi-omics integrated clustering algorithms, we recognized two distinct subtypes and determined their respective numbers (Fig. 2A and B). Subsequently, we integrated molecular expression patterns across various transcriptomes (including mRNAs, lncRNAs, and miRNAs), epigenetic methylation patterns, and somatic mutations using a consensus-based approach (Fig. 2C and E). Our classification system demonstrated a strong correlation with overall survival (OS) (*p* = 0.011; Fig. 2F). Significantly, CS1 showed the most favorable survival outcome among all identified subtypes.

### Validation of molecular subtypes

**Fig. 3:**
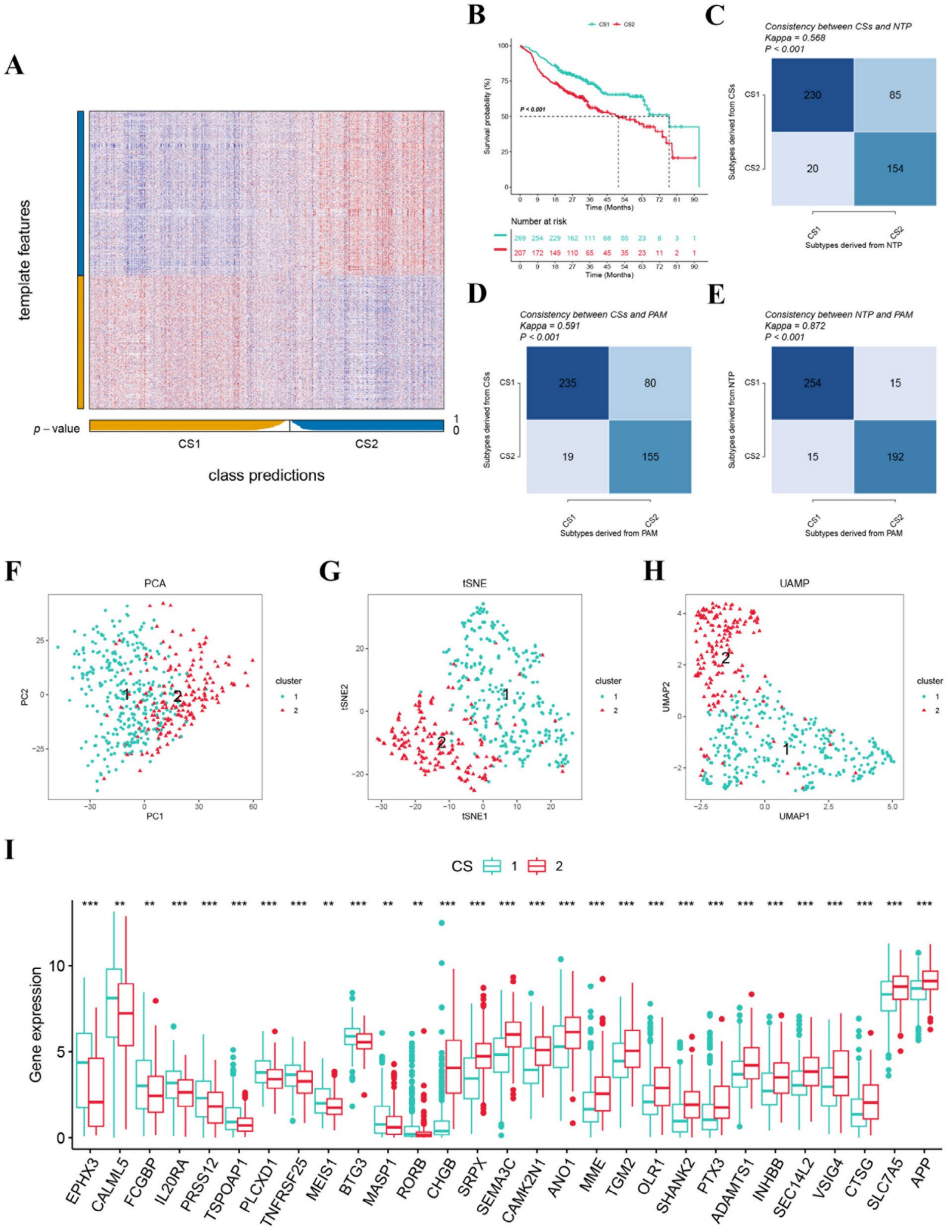
Molecular landscape for different CSs. (**A**) Validation of molecular subtypes in the META-HNSCC dataset; (**B**) Survival analysis in the META-HNSCC dataset; (**C**) The consistency between CSs and NTP in the TCGA-HNSCC dataset; (**D**) The consistency between CSs and PAM in the TCGA-HNSCC dataset; (**E**) The consistency between NTP and PAM in the META-HNSCC dataset; (**F**–**H**) PCA, tSNE, and UMAP validated two clusters. (**I**) The expression patterns in the various CSs

We identified 1000 genes uniquely overexpressed in each subtype as classifiers. These genes were subsequently validated in an external cohort to confirm the stability of the identified subtypes. Comparable results were observed in the META-HNSCC cohort (Fig. 3A). Notably, the CS1 subtype within the META-HNSCC cohort, which encompassed three datasets, exhibited the most favorable prognosis among all subtypes (*p* < 0.001; Fig. 3B). Additionally, the NTP successfully categorized each sample in the external cohort into the previously identified CSs. The consistency of the CSs with both the NTP and PAM methods was further evaluated and found to be statistically significant (*p* < 0.001; Fig. 3C and E). PCA, tSNE and UMAP methods showed significant differences in mRNA expression profiles between groups (Fig. 3F and H).

### Gene expression profile, enrichment analysis, and immune cell infiltration

**Fig. 4:**
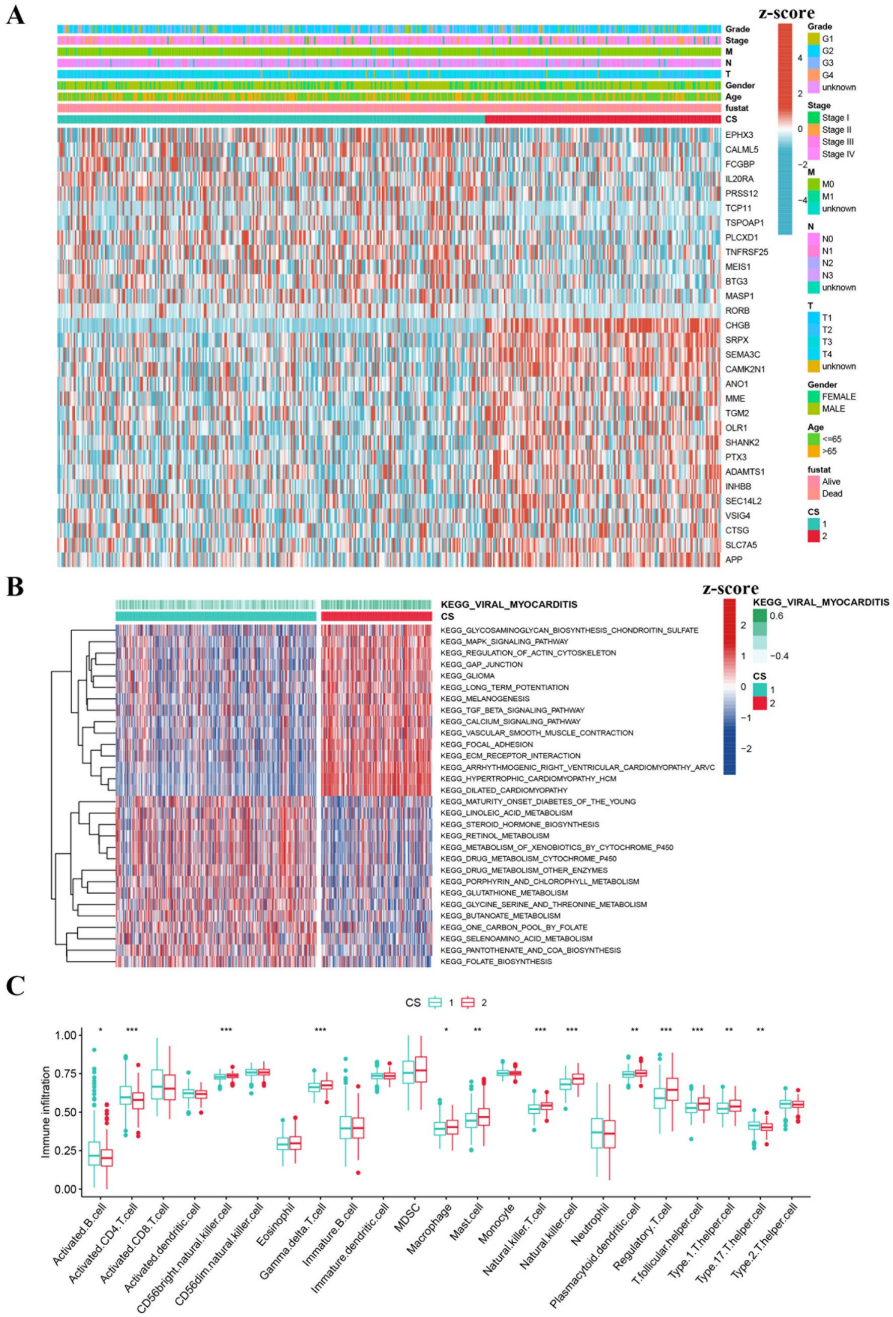
Distinct clinical features and immune landscapes of HNSCC CSs. (**A**) Heatmap of the clinical and pathological characteristics of the two clusters; (**B**) The heatmap of KEGG pathways of the two clusters; (**C**) Abundances of infiltrating immune cells

CS2 showed considerably higher expression levels of genes in the follow-up model compared to CS1, indicating a stronger association between genes and CS2 (Fig. 3I). Clinical analysis showed that CS2 was associated with greater age, females, non-metastasis, and other clinical information (Fig. 4A). The results indicated that CS1 was enriched in pathways related to glycosaminoglycan biosynthesis, chondroitin sulfate, MAPK signaling pathway, regulation of actin cytoskeleton, gap junction, and glioma. CS2 was significantly enriched in pathways related to maturity onset diabetes of the young, linoleic acid metabolism, steroid hormone biosynthesis, retinol metabolism, and metabolism of xenobiotics by cytochrome p450 (Fig. 4B). The ssGSEA algorithm yielded a higher level of immune cell infiltration in CS2 (Fig. 4C).

### Development of the machine learning-driven signature

**Fig. 5:**
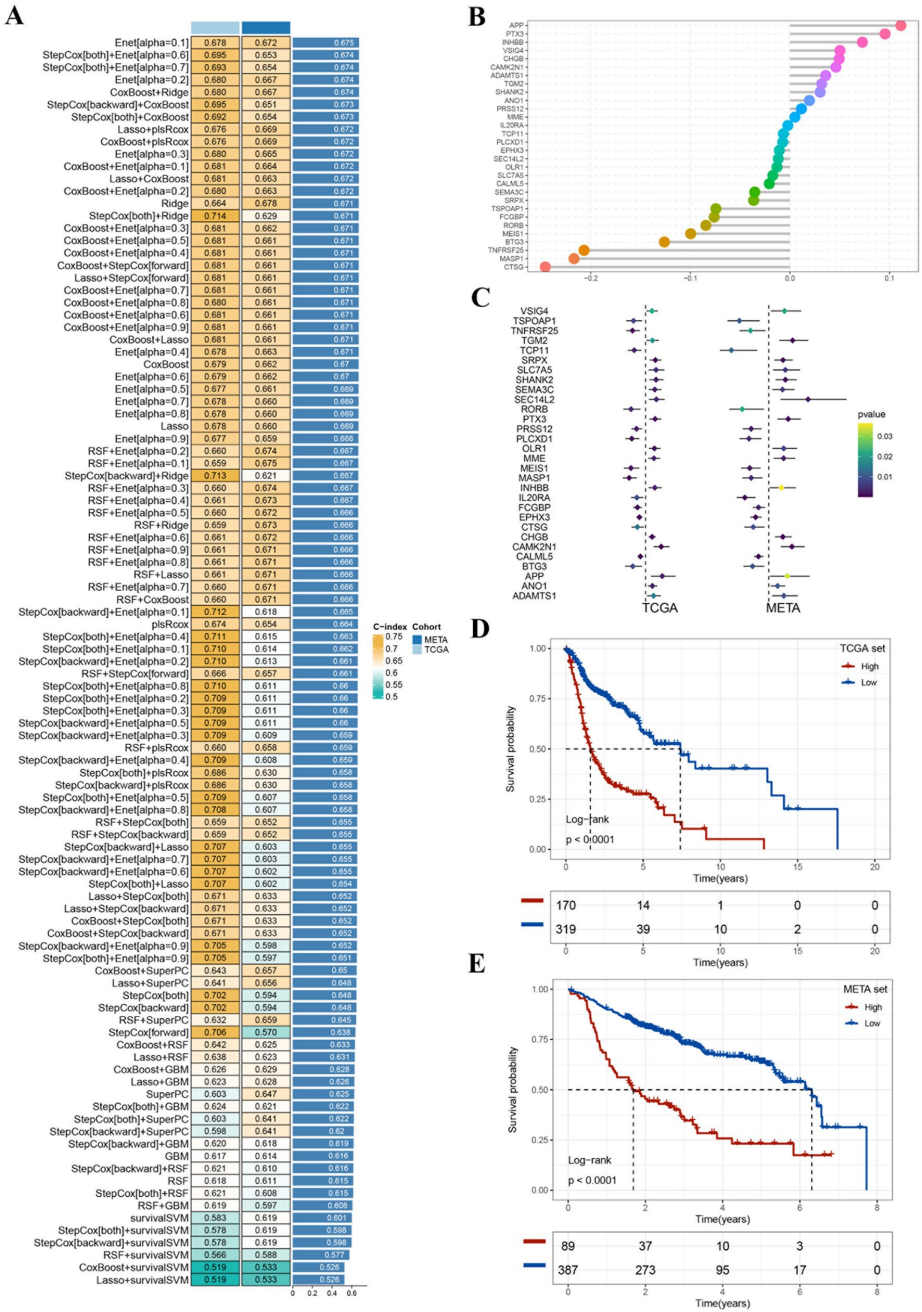
Construction of the prognostic signature. (**A**) C-index values of 101 machine learning algorithms in TCGA-HNSCC and META-HNSCC cohorts; (**B**) Selection of hub PRGs by the Enet [alpha = 0.1] algorithm; (**C**) Univariate Cox analysis of hub PRGs in TCGA-HNSCC and META-HNSCC cohorts; (**D**–**E**) Survival analysis of different groups in the TCGA-HNSCC and META-HNSCC cohorts

The development pipeline of the machine learning-driven signature is as follows. First, we performed univariate Cox analysis within the TCGA-HNSCC and META-HNSCC datasets. Genes with *p* < 0.05 and the same HR orientation were considered PRGs. We screened 135 PRGs from the TCGA-HNSCC and META-HNSCC cohorts, and their expression was significantly associated with OS. Among them, 12 PRGs were risk factors, while 18 PRGs were protective factors. Then, 101 combinations of 10 machine learning algorithms were utilized to develop the most predictive signature with the best C-index performance. Finally, upon establishing the model on the training set, we proceeded to rigorously test it across all validation cohorts. For each model, we computed the average C-index, ultimately considering the model with the highest C-index value as the optimal one (Fig. 5A). The model incorporating Enet [alpha = 0.1] demonstrated the highest average C-index and was chosen to construct the final model, which was based on 30 PRGs (Fig. 5B and C). Subsequently, we calculated risk scores for individual samples in all cohorts. Notably, the high-risk group within both the TCGA-HNSCC and META-HNSCC sets displayed a less favorable clinical outcome (Fig. 5D and E). To validate the prognostic significance of the genes included in the model, we employed Kaplan-Meier analysis in HNSCC. These results were largely consistent with those derived from the Cox analysis (Fig.S2 and S3). Our findings indicated a significant association of these genes with DSS and PFS in HNSCC, underscoring their strong prognostic relevance to patient outcomes (Fig. S4 and S5).

### Comparison of prognostic signatures

**Fig. 6:**
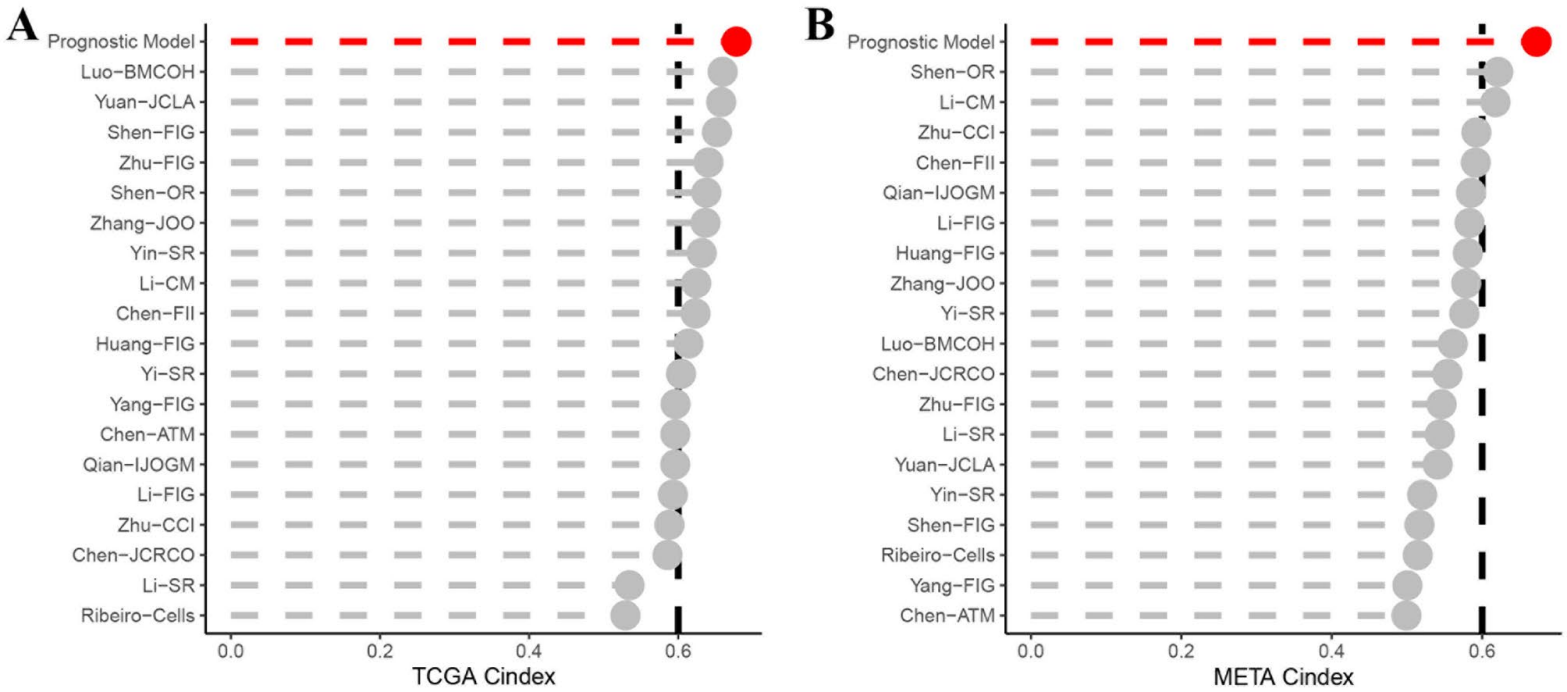
(**A**–**B**) Comparison between the prognostic signature and the 17 published signatures in the TCGA and META cohorts

**Fig. 7:**
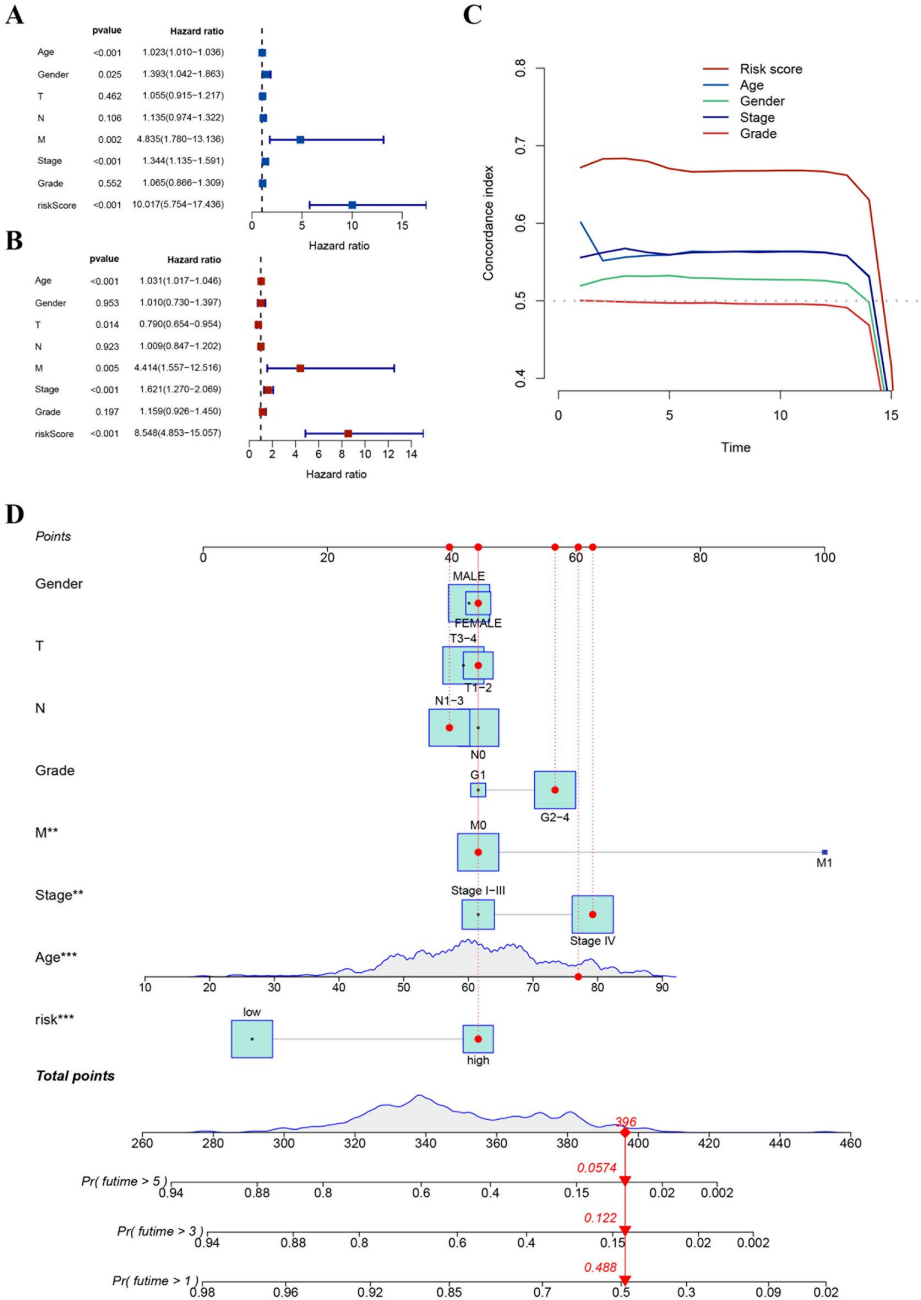
Construction of independent prognostic analyses and a clinical nomogram. (**A**) Univariate Cox analyses for the prognostic and clinical features; (**B**) Multivariate Cox analyses for the prognostic and clinical features of patients in the TCGA-HNSCC dataset; (**C**) C-index analysis of prognostic accuracy in the TCGA-HNSCC dataset; (**D**) Nomogram construction of the model and clinicopathological characteristics for HNSCC

In order to conduct a comparison between the prognostic signature and others, we examined 17 different published models. These published models are related to various biological processes, such as angiogenesis, hypoxia, pyroptosis, circadian regulation, fatty acid metabolism, necroptosis, immune response, ferroptosis, etc. Remarkably, the prognostic signature exhibited superior C-index performance compared to all models in both the TCGA-HNSCC and META-HNSCC sets (Fig. 6A and B). Univariate and multivariate Cox analyses confirmed that the risk score derived from the signature was an independent prognostic factor (Fig. 7A and B). Furthermore, the C-index analysis validated the enhanced prognostic efficacy of the signature over clinical characteristics (Fig. 7C). To enable accurate prediction of HNSCC survival, a nomogram was developed integrating the prognostic model and clinical characteristics (Fig. 7D). These findings collectively underscore the robust predictive power and clinical utility of our prognostic signature in the context of HNSCC, positioning it as a valuable tool for informing patient outcomes and treatment strategies.

### Exploration of molecular functions, pathways, and gene mutations

**Fig. 8:**
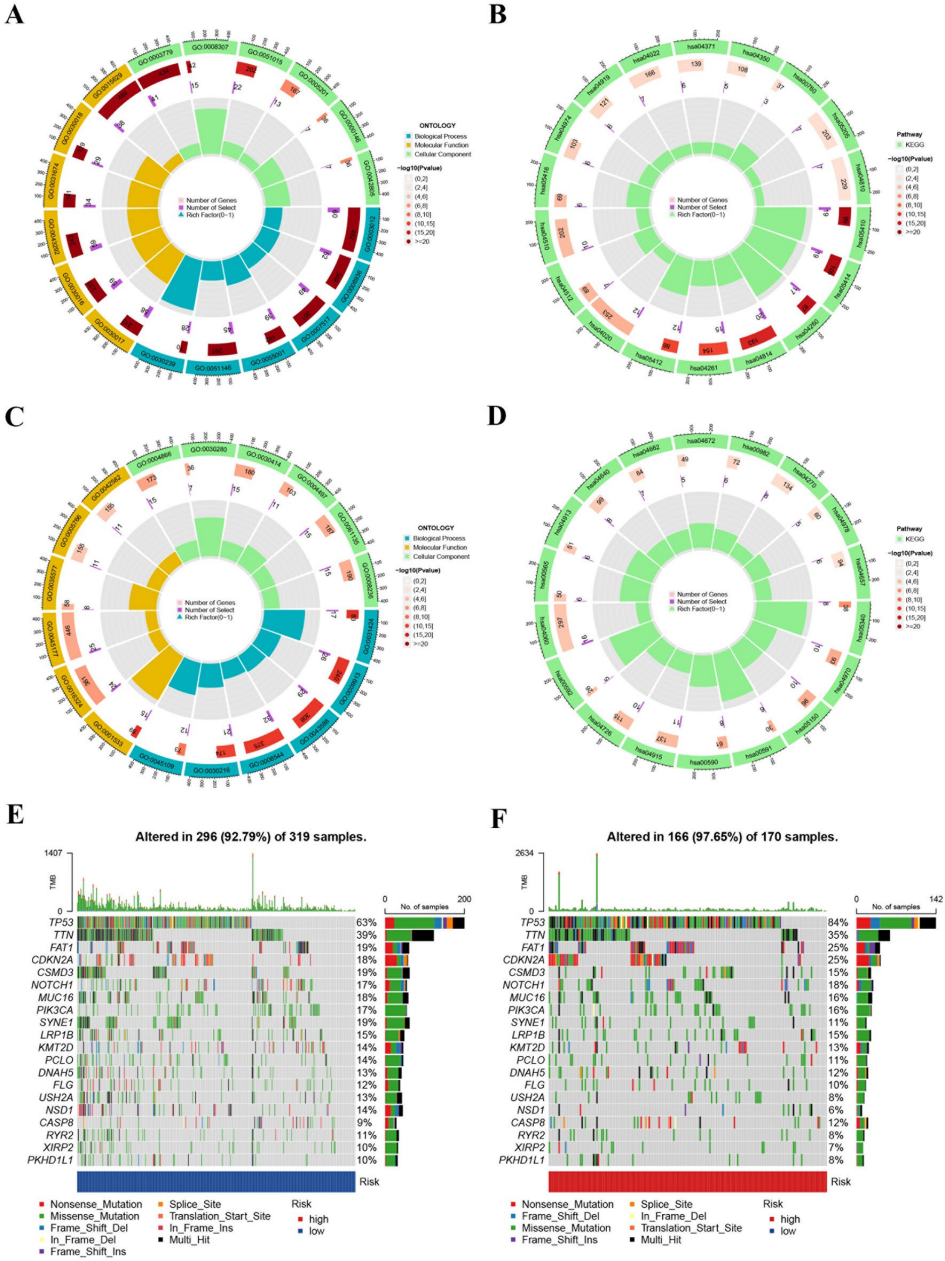
Functional enrichment analysis and gene mutation in HNSCC. (**A**–**D**) Circle plot for GO and KEGG analysis; (**E**–**F**) Gene mutation patterns between patients with different risk groups

704 DEGs were recognized among various groups. BP terms for the high-risk group were related to muscle system processes, muscle contraction, and muscle organ development. CC terms for the high-risk group were associated with sarcomere, myofibril, and contractile fiber. MF terms for the high-risk group were related to actin binding, structural constituents of muscle, and actin filament binding (Fig. 8A and Table S1). KEGG analysis indicated that the high-risk group was enriched in pathways including hypertrophic cardiomyopathy, dilated cardiomyopathy, cardiac muscle contraction, motor proteins, and adrenergic signaling in cardiomyocytes (Fig. 8B and Table S2). The molecular functions and pathways of the high-risk group mainly focused on cell-molecule interactions, metabolic and uptake processes, and immune and inflammatory responses. BP terms for the low-risk group were related to keratinization, epidermal cell differentiation, and skin development. CC terms for the low-risk group were related to the cornified envelope, apical plasma membrane, and apical part of the cell. MF terms for the low-risk group were related to endopeptidase inhibitor activity, structural constituents of skin epidermis, and peptidase inhibitor activity (Fig. 8C and Table S3). KEGG analysis showed that the low-risk group was enriched in pathways including primary immunodeficiency, salivary secretion, staphylococcus aureus infection, linoleic acid metabolism, and arachidonic acid metabolism (Fig. 8D and Table S4). The molecular functions and pathways in the low-risk group were mainly focused on immunity and infection, metabolism and nutrition, endocrinology, and signaling. Moreover, the top 10 genes with mutations were identified, and the frequency of mutations was higher in the high-risk group (Fig. 8E and F).

### Exploration of the immune landscape

**Fig. 9:**
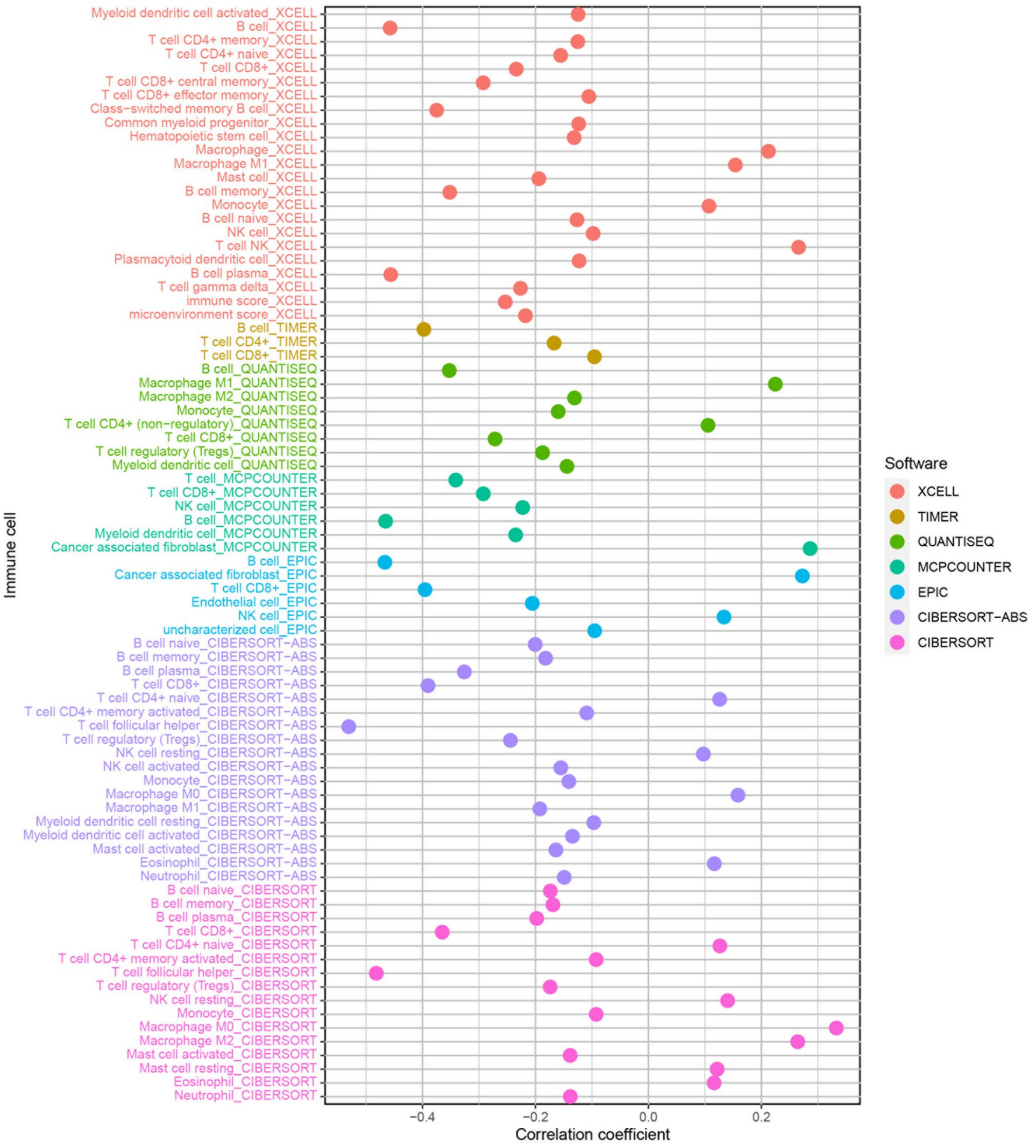
The immune cells within the TME in HNSCC

**Fig. 10:**
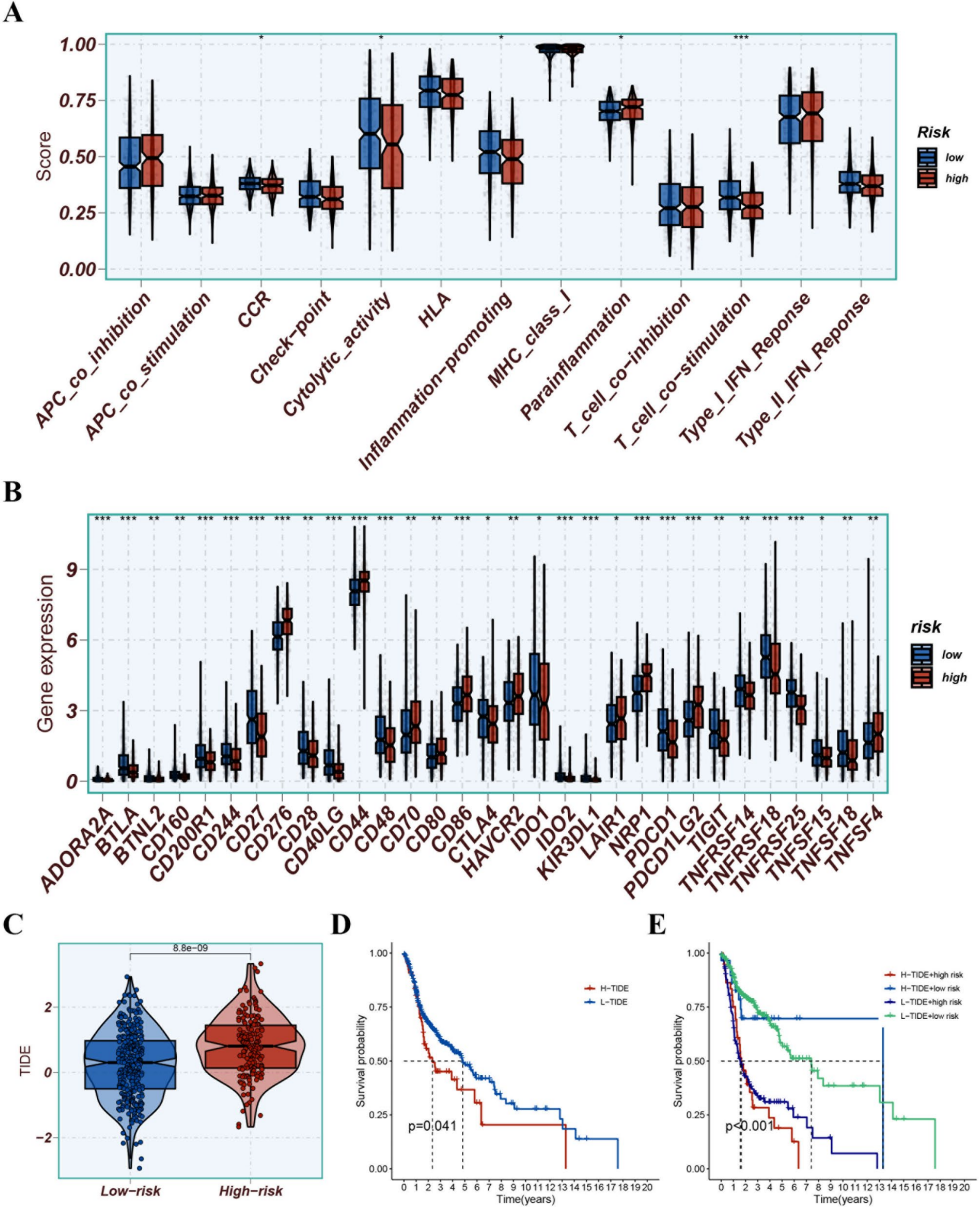
The TME-related molecular characteristics of various groups. (**A**) Comparison of immune function between various groups; (**B**) Difference the expression levels of ICGs in the two groups; (**C**) The low-risk patients demonstrated markedly lower TIDE scores; (**D**) Survival probability between H-TIDE and L-TIDE; (**E**) Survival probability between H-TIDE + high risk, H-TIDE + low risk, L-TIDE + high risk, and L-TIDE + low risk

The risk score was negatively connected with B cells, mast cells, monocytes, myeloid dendritic cells, neutrophils, and T cells (Fig. 9). Some immune functions were activated in the low-risk group, including CCR, cytolytic activity, inflammation-promoting, and T cell co-stimulation (Fig. 10A). Expression of ICGs was also higher in the low-risk group, including CTLA4, PDCD1, TIGIT, and IDO1 (Fig. 10B). The low-risk patients had markedly lower TIDE scores, indicating a more sensitive response to immunotherapy (*p* < 0.001; Fig. 10C). Finally, we found that combining the TIDE score and risk score could better predict patient prognosis (Fig. 10D and E).

### Identification of drugs

**Fig. 11:**
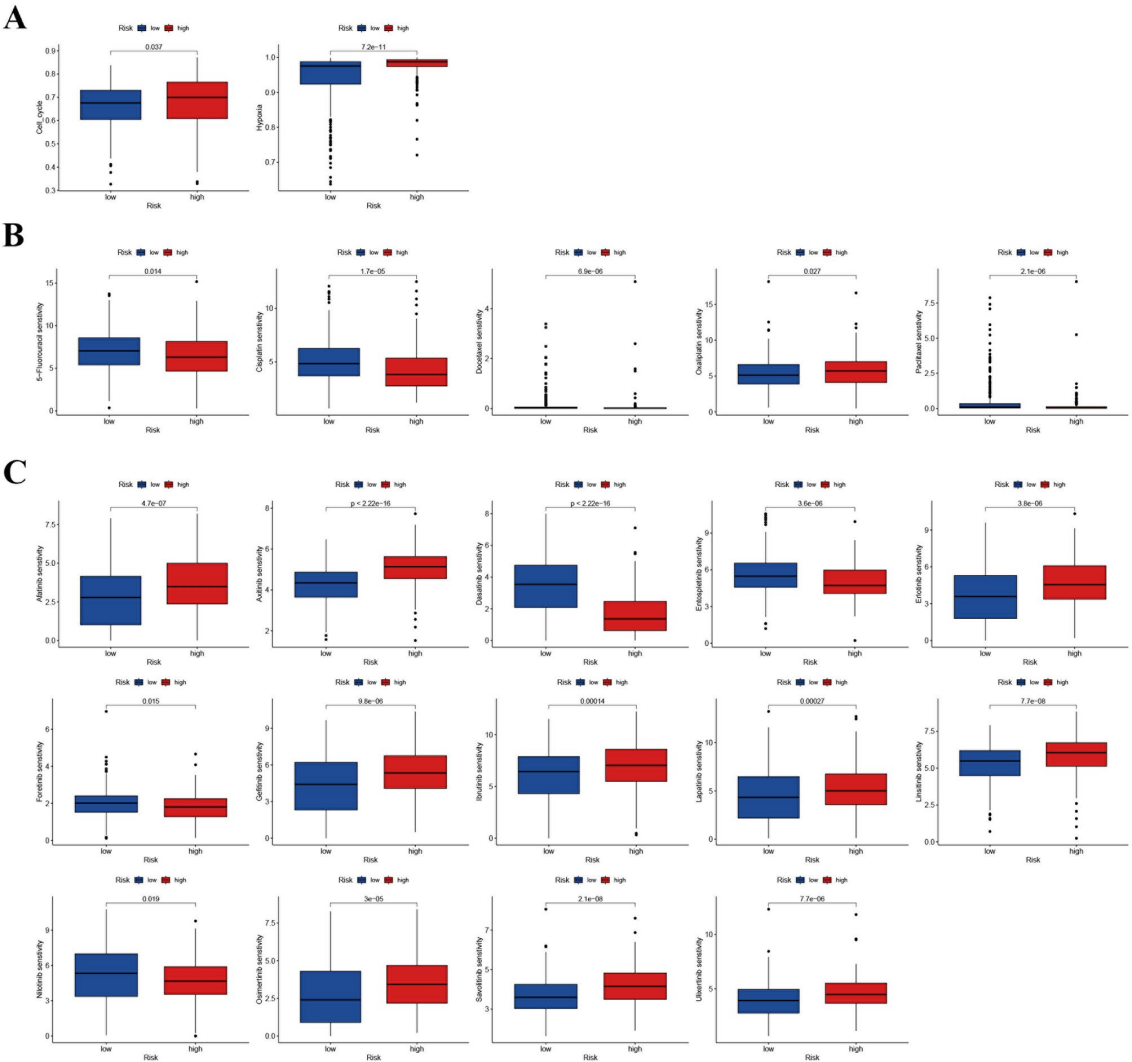
Comparison of drug sensitivity between different groups. (**A**) Two radiotherapy-associated biomarkers (cell cycle and hypoxia) were enriched in the high-risk group; (**B**) Comparison of drug sensitivity (5-Fu, Cisplatin, Docetaxel, and Paclitaxel) between various groups; (**C**) The drug sensitivity of targeted therapies between different groups

Two radiotherapy-associated biomarkers (cell cycle and hypoxia) were markedly enriched in the high-risk patients, suggesting greater suitability for radiation treatment (Fig. 11A). Four commonly used chemotherapeutic drugs were more sensitive in the high-risk group, implying a greater likelihood of benefit from chemotherapy (Fig. 11B). Most EGFR antagonists were more sensitive in the low-risk group, indicating that targeted EGFR therapy is more appropriate for low-risk patients (Fig. 11C).

### Exploration of scRNA

**Fig. 12:**
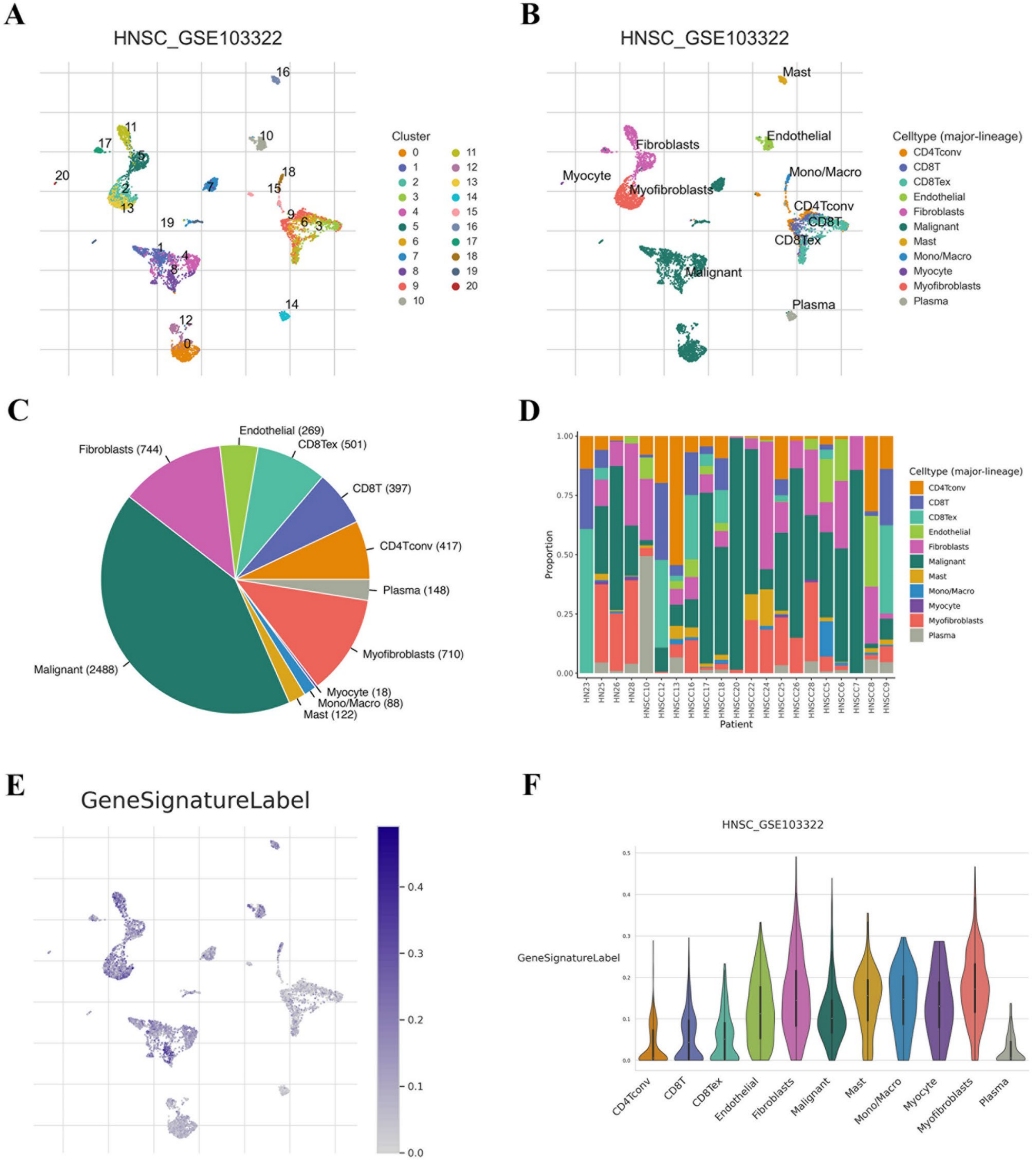
The immune microenvironment and cellular communication characteristics of HNSCC at the single-cell level. (**A** and **B**) The distribution of various clusters and cell types from the GSE103322 cohort; (**C** and **D**) The proportion of different cell types in various samples; (**E**) The signature expression levels were significantly higher in myo-fibroblasts, fibroblasts, and mast cells in the GSE103322 cohort

The GSE103322 dataset contains approximately 5,902 single cells from 18 HNSCC patients, including 5 matched pairs of primary tumors and lymph node metastases. Figure 12A illustrated the distribution of 20 distinct clusters; Fig. 12B displayed the distribution of 11 various cell types; Fig. 12C presented the proportions of these 11 cell types; and Fig. 12D demonstrated the proportions of the 11 cell types across different patient samples. Notably, the expression levels of genes in the model were significantly elevated in myofibroblasts, fibroblasts, and mast cells (Fig. 12E and F).

### Quantitative RT-PCR analysis

We detected the expression levels of 11 PRGs (APP, PTX3, INHBB, VSIG4, CHGB, CAMK2N1, ADAMTS1, TGM2, SHANK2, ANO1, PRSS12) in control and tumor cell groups using qRT-PCR analysis. The results showed that the mRNA expression levels of 10 PRGs (APP, PTX3, INHBB, VSIG4, CAMK2N1, ADAMTS1, TGM2, SHANK2, ANO1, PRSS12) were upregulated in the tumor group (Fig. S6).

## Discussion

The rapid advancement of high-throughput sequencing technologies has driven advances in oncological research and helped us gain deeper knowledge of the intrinsic mechanisms and mutational features of tumorigenesis [67]. Zhu et al. analyzed the sequencing data of HNSCC from the GEO and TCGA databases to identify the potential role of the pyroptosis-related gene [38]. Yin et al. used cell differentiation trajectories to identify HNSCC molecular classifications and gene signatures that forecast prognosis and immunotherapy response, offering more integrated predictions and perspectives for the treatment of HNSCC [36]. Zhu et al. constructed an inflammation-related model for HNSCC to predict survival outcomes and treatment response in patients by analyzing transcriptomic data from HNSCC patients, providing a new entry point and direction for the investigation of HNSCC-related immunotherapy [46]. However, single-omics analyses make it difficult to perform in-depth investigations in the context of complex biological mechanisms and have limited reliability and persuasive power for research conclusions [68]. By analyzing these histological data together, researchers are able to capture key biological signals that may have been missed in a single histological analysis, resulting in a more comprehensive understanding of the complex immune response mechanisms of disease. This approach can reveal interactions between different histological dimensions and help identify potential immune escape mechanisms or specific immunotherapeutic targets [69]. Machine learning algorithms offer several advantages over traditional algorithms, including the ability to handle complex and non-linear relationships, adaptability to changing conditions, efficient processing of large-scale data, and superior generalization capabilities [70]. Therefore, our study adopted multi-omics integrated analysis to produce important evidence for the essential basis for precision and individualized treatment of HNSCC.

In this research, we recognized CSs (CS1 and CS2) from 10 multi-omics algorithms, and CS1 indicated the most favorable survival outcome. CS1 also proved to have the best prognosis in the META-HNSCC cohort. The level of immune cell infiltration was significantly higher in CS2, suggesting that CS2 patients were more sensitive to immune responses. We also screened 135 PRGs from the TCGA-HNSCC and META-HNSCC cohorts using univariate Cox analysis. We further screened 30 PRGs to be included in the study to obtain a prognostic signature and construct the integration framework. Based on 101 algorithms, we computed the average C-index for each model to evaluate its predictive power. And risk scores revealed that the high-risk patients might suffer from a worse clinical prognosis. Compared to 17 other published signatures, the prognostic signature displayed strong predictive power in each of the cohorts. We performed functional enrichment analyses of DEGs from various groups and found that multiple cancer-related pathways were significantly activated in the high-risk group, indicating that they were more susceptible to cancer development. Besides, the top 10 mutated genes had higher frequencies in the high-risk group.

We screened 135 PRGs from the TCGA-HNSCC and META-HNSCC cohorts, and their expression was significantly associated with OS. Among them, 12 PRGs were risk factors, while 18 PRGs were protective factors. Consistent with previous research, these PRGs have been shown to be significantly associated with prognosis and biological functions across a variety of tumor types, including HNSCC [71–74]. APP is an androgen-induced gene that promotes the proliferative activity of breast cancer cells and has been recognized as a potent prognostic factor in patients with ER-positive breast cancer [75]. PTX3 overexpression accelerates tumor metastasis and suggests poor prognosis in hepatocellular carcinoma by driving epithelial-mesenchymal transition [76]. VSIG4 expression is associated with poor prognosis in patients with progressive gastric cancer [77]. High ADAMTS1 expression is associated with reduced survival in patients with lymph node-negative breast cancer [78]. High TGM2 expression in LUSC is associated with poorer prognosis, and high TGM2 expression is strongly associated with pro-tumor inflammation and may increase susceptibility to immunotherapy [79]. Enhanced expression of ANO1 in HNSCC leaded to cell migration and is associated with poor prognosis [80]. CTSG inhibited proliferation and metastasis of HNSCC by blocking the JAK2/STAT3 pathway [81]. Overexpression of MASP1 in hepatocellular carcinoma cell lines significantly inhibited proliferation, invasion and migration [82]. BTG3 protein expression might be considered as a good marker indicating good prognosis in epithelial ovarian carcinoma [83]. MEIS1 was downregulated in most tumors, and high MEIS1 expression predicted better OS in patients with HNSCC, adrenocortical carcinoma, and clear cell renal carcinoma [84]. These findings collectively highlight the intricate roles that PRGs play in modulating tumor biology and prognosis, providing valuable insights for future therapeutic strategies and prognostic assessments.

By using the ssGSEA algorithm and the TIDE algorithm, we analyzed the immunization status between various groups. Some immune function was activated in the low-risk patients, and the expression of ICGs (CTLA4, PDCD1, TIGIT, and IDO1) was high in the low-risk patients. CTLA4 is a protein receptor that plays a vital role in regulatory T cell activation and tolerance [85]. The immunosuppressive effects of CTLA-4 effectively stimulate the immune response, thereby affecting the proliferation of cancer cells. Ipilimumab, as a CTLA-4 inhibitor, was the first drug proven to prolong OS in patients with advanced melanoma [86]. PDCD1 (PD-1) can be expressed on the surface of immune cells [87]. PD-1 regulates the immune system by down-regulating the immune response to human cells and inhibiting T-cell inflammatory activity [88]. Pembrolizumab (PD-1 antibodies) as a first-line treatment strategy for HNSCC was shown to improve the prognosis of advanced HNSCC [89]. TIGIT is a suppressor receptor shared by T and NK cells that suppresses tumor cell killing by NK and T cells [90]. TIGIT could lead to NK cell depletion during tumor progression, and further studies revealed that an anti-TIGIT monoclonal antibody could reverse NK cell depletion and be used in immunotherapy for a variety of tumors [91]. In addition, based on the results of available clinical studies, anti-TIGIT antibody drugs are regarded as enhancing the human immune response against cancer cells [92]. IDO1 is the rate-limiting enzyme for the conversion of metabotropic tryptophan to kynurenine [93]. Overexpression of IDO1 in tumor tissues caused tryptophan depletion in the TME, which suppressed T-cell immune function and mediated immune escape from tumors [94]. IDO inhibitors (including Epacadostat and Indoximod) are currently in clinical trials and are expected to be used in the future as molecular immunotherapeutic agents for tumors in the treatment of cancer [95]. Moreover, the low-risk patients demonstrated markedly lower TIDE scores, demonstrating more sensitivity to immunotherapy. The above findings suggest that immunotherapy may benefit the low-risk group.

The mainstays of clinical treatment for HNSCC included surgery, radiotherapy, and chemotherapy. The majority of advanced HNSCC patients could be eradicated by surgery; however, some patients still needed to be treated with chemotherapy to control the progression of the disease after surgery [96]. We found that radiotherapy, 5-fluorouracil (5-Fu), Cisplatin, Docetaxel, and Paclitaxel were more sensitive in the high-risk group. 5-Fu is an antimetabolite broad-spectrum antitumor agent that is widely used as the classical chemotherapeutic agent for multiple malignancies [97]. As a first-line anticancer drug, cisplatin is employed in the treatment of various solid tumors, but studies showed that cisplatin had significant toxic effects on the kidneys, limiting its use in clinic treatment [98]. Since 2010, taxane-based anticancer drugs have been applied to the treatment of HNSCC, such as docetaxel and paclitaxel [99]. Despite greater advances in the treatment of HNSCC with chemotherapy, it did not prolong the OS of patients, and the prognosis is still poor. At the present stage, targeted drug therapy has demonstrated great therapeutic potential in HNSCC [100]. Our further analyses revealed that targeted therapies were more sensitive in low-risk patients. High EGFR expression is related to a worse survival condition in HNSCC patients [101]. Zalimumab is a monoclonal antibody against EGFR that effectively inhibits tumor growth by blocking EGFR signaling in preclinical models [102]. Cetuximab, an anti-EGFR monoclonal antibody, combined with radiotherapy, was found to improve OS in some patients with locally advanced HNSCC [103].

Fibroblasts, particularly cancer-associated fibroblasts (CAFs), are critical components of the TME and play pivotal roles in the genesis and progression of HNSCC. Signaling molecules such as TGF-β, FGF, and VEGF secreted by CAFs can activate signaling pathways in HNSCC tumor cells and promote cell division and growth. CAFs can also attract endothelial cells to enter the HNSCC tumor microenvironment through the secretion of chemokines to further promote angiogenesis. CAFs also enhance the migration ability and invasiveness of tumor cells by regulating EMT processes in HNSCC tumor cells. Immunomodulatory factors such as TGF-β and IL-6 secreted by CAFs can inhibit the activity of effector T cells and promote immunosuppressive cells such as regulatory T cells. migratory ability and invasiveness of tumor cells. Immunomodulatory factors such as TGF-β and IL-6 secreted by CAFs inhibit the activity of effector T cells and promote the accumulation of immunosuppressive cells such as regulatory T cells and myeloid-derived suppressor cells.By altering the physical properties of the tumor microenvironment, such as increasing the density of the extracellular matrix, CAFs limit the penetration of drugs into tumor tissues and reduce the effects of chemotherapy drugs and the killing effect of radiotherapy on tumor cells. These effects include promotion of tumor proliferation and growth, promotion of tumor angiogenesis, promotion of tumor metastasis and invasion, modulation of immune escape, and promotion of resistance to chemotherapy and radiotherapy [104]. Analysis of scRNA-seq indicated that the expression of the genes within our model was significantly upregulated, predominantly in fibroblasts. This suggests that these genes may further influence tumor development through their interaction with fibroblasts.

Our study has several limitations that merit careful consideration. First, the retrospective design relying on pre-existing datasets (e.g., TCGA, GEO) inherently carries risks of selection bias. Since these data were derived from specific institutions, regions, or subpopulations, the cohorts may lack representativeness across diverse geographic, ethnic, or clinical contexts. This compromises the external validity of our findings and limits their generalizability to broader clinical settings. Second, incomplete or inconsistently annotated clinical data in public repositories, such as treatment histories or HPV status, restricted our ability to perform granular subgroup analyses. Such gaps could introduce systematic bias, potentially leading to overinterpretation of results and misalignment with real-world patient outcomes. Third, while our computational models identified robust molecular subtypes and prognostic signatures, the biological mechanisms underlying these findings remain speculative. Experimental validation—via functional assays or prospective clinical cohorts—is essential to confirm the causal roles of the 30 PRGs in HNSCC progression and therapy resistance.

Despite these limitations, our conclusions are strengthened by rigorous cross-validation using independent cohorts (e.g., META-HNSCC), which confirmed the reproducibility of the subtypes (CS1/CS2) and the prognostic signature. Clinicians should interpret these results cautiously, prioritizing supplementary validation in localized patient populations before implementing risk-stratified therapies. Moving forward, prospective multi-center studies with standardized data collection protocols and integrated multi-omics profiling will be critical to refine these models and translate them into actionable clinical tools.

## Conclusion

In conclusion, we identified two CSs of HNSCC using multi-omics data, predicted the prognosis and treatment response of patients by constructing a model with 30 PRGs and the Enet [alpha = 0.1] algorithm, and finally found that the high-risk patients were more sensitive to radiotherapy, 5-Fu, cisplatin, doxorubicin, and paclitaxel, whereas the low-risk patients were more sensitive to immunotherapy and targeted therapies. These findings provided valuable insights into the diagnosis and personalized treatment of HNSCC patients and offered a promising new strategy for clinical practice.

**Table 1:** Relevant information about the dataset

Datasets	Sample	Experiment type	Annotation platform	Omics data
TCGA-HNSCC	489	High throughput sequencing	-	Transcriptome, methylationome, mutagenome, and clinical data
GSE27020	109	Microarray	GPL96	Transcriptome, and clinical data
GSE41613	97	Microarray	GPL570	Transcriptome, and clinical data
GSE65858	270	Microarray	GPL10558	Transcriptome, and clinical data

## Electronic supplementary material

Below is the link to the electronic supplementary material.

Supplementary Material 1

Supplementary Material 2

Supplementary Material 3

Supplementary Material 4

Supplementary Material 5

Supplementary Material 6

Supplementary Material 7

## Data Availability

All results generated in this study can be obtained by contacting the corresponding authors on reasonable request. The complete code and critical data are available on Github (https://github.com/JYfantast/HNSCC).
