# Supplementary figures and images for: Multiple machine learning-based integrations of multi-omics data to identify molecular subtypes and construct a prognostic model for HNSCC
Source: Hereditas. 2025 Feb 6;162:17. doi: 10.1186/s41065-025-00380-0 (PMC11800565; doi:10.1186/s41065-025-00380-0)

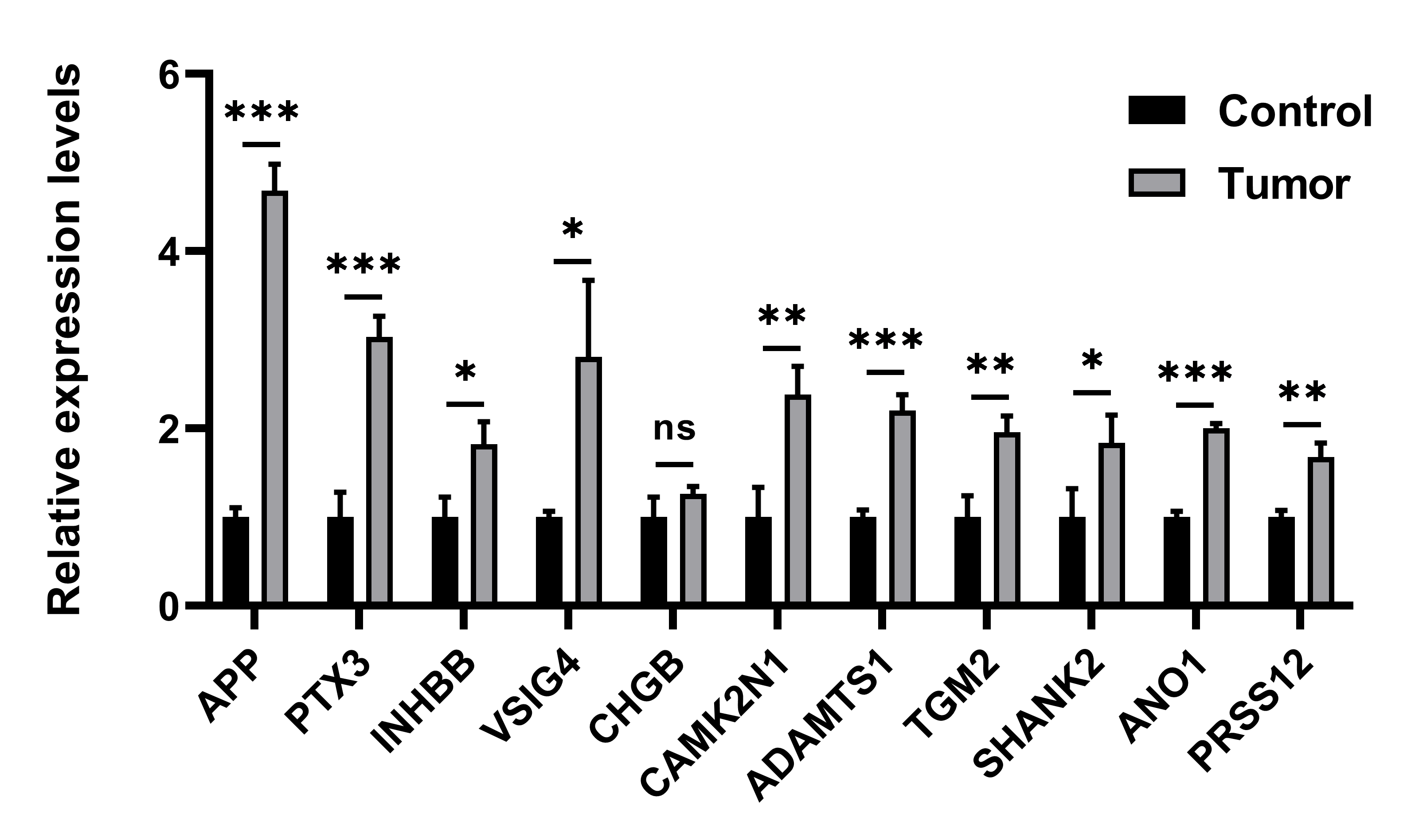

Supplement: Supplementary file 3 — Supplementary Material 3 [file 41065_2025_380_MOESM3_ESM.tif]
